# Understanding the Autistic Experience of Restrictive Eating Disorders—A Systematic Review and Qualitative‐Synthesis

**DOI:** 10.1002/erv.3181

**Published:** 2025-03-05

**Authors:** Rachel Loomes, Katy Chivers, Chloé Georgeaux‐Healy, Will Mandy, Tom Jewell

**Affiliations:** ^1^ Department of Clinical, Educational & Health Psychology University College London London UK; ^2^ Children & Young Persons' Community Eating Disorder Service South West London & St George's Mental Health NHS Trust London UK; ^3^ Florence Nightingale Faculty of Nursing, Midwifery & Palliative Care King's College London London UK; ^4^ The Bethlem Royal Hospital South London and Maudsley NHS Foundation Trust London UK; ^5^ Psychological and Mental Health Services Great Ormond Street Hospital NHS Foundation Trust London UK

**Keywords:** anorexia nervosa, autism, qualitative, systematic review, treatment

## Abstract

**Objective:**

To synthesise qualitative findings on the autistic experience of restrictive eating disorders in order to identify common themes and use this to inform future research on the development of more effective care.

**Method:**

This systematic review was pre‐registered on PROSPERO (CRD42023434116) and followed PRISMA guidelines. CINAHL, PsycINFO, Medline, Embase, Web of Science and Global Health databases were searched. Studies were included if they contained qualitative data detailing the autistic experience of restrictive eating disorders from autistic people, carers or healthcare professionals. The Critical Appraisal Skills Programme was used to assess quality of studies. Recurring themes were identified via thematic synthesis.

**Results:**

Nine studies met the inclusion criteria, all focused on anorexia nervosa. Four themes arose from the analysis: (1) the relationship between autism and restrictive eating; (2) the journey to self‐understanding; (3) experience of eating disorder services; (4) suggested treatment adaptations.

**Conclusion:**

Findings suggest a broad range of mechanisms underlying the development and perpetuation of anorexia nervosa that are related to autism and are not consistently acknowledged and addressed in current care provision. This emphasises the need for more research into developing adapted or novel interventions for autistic people with eating disorders, as well as training programmes for clinicians.

**Summary**

- Autistic people, parents and healthcare professionals consistently report that autism plays a significant role in the development and maintenance of anorexia nervosa.
- These autism‐related difficulties are not reliably integrated into the formulation and treatment of the eating disorder. To correct this there is a need to develop autism‐adapted treatments alongside training programmes for clinicians. Both should be co‐produced with people with lived experience of autism and restrictive eating disorders.
- The systematic search identified no qualitative studies with either autistic children or adolescents with anorexia nervosa, as well limited representation of males and non‐binary people. Studies did not consistently report on ethnicity. There is a need for qualitative research to capture the experiences of these populations.

## 1 Introduction

Eating Disorders (ED) are characterised by a persistent disturbance of eating or eating‐related behaviours resulting in impaired physical and psychosocial functioning (APA 2013). EDs have the highest reported mortality rates among mental disorders, and risk of physical complications and mental health comorbidity are high (Arcelus et al. 2011; Chesney, Goodwin, and Fazel 2014). Previous research has sub‐categorised the eating disorders into restrictive (RED) and non‐restrictive eating disorders (Brede et al. 2020). REDs are further parsed into Anorexia Nervosa (AN), and certain presentations of Other Specified Feeding or Eating Disorder (OSFED), such as atypical AN.

Autism is a neurodevelopmental condition characterised by socio‐communicative difficulties, presence of restricted interests and patterns of behaviour, and differences in sensory processing (APA 2013). Autism presents heterogeneously, with high mental health comorbidity (Lai, Lombardo, and Baron‐Cohen 2014), including EDs (Westwood and Tchanturia 2017). Please note that we use identity‐first language (e.g. autistic person) rather than person‐first language (e.g. person with autism). This is based on research findings on the preferences of the autistic community (Bury et al. 2023; Kenny et al. 2016).

Quantitative research has highlighted that long term outcomes of REDs, such as AN, are generally poorer for people who are autistic or have a high level of autistic traits (Nielsen et al. 2015; Tchanturia et al. 2019). There is therefore a need for adapted or novel therapeutic interventions that are tailored to the distinctive challenges that autistic people face when experiencing a RED, as well as harnessing the strengths of being autistic to promote change. In order to develop these it is important that any proposed changes to treatment are centred around the lived experience of autistic people and people who support them, both in terms of family/carers and healthcare professionals. They are likely to have insights based on their lived experience about why current service provision for REDs are not effective for them, as well as ideas about solutions. Qualitative research can contribute to this understanding by providing richer and more detailed accounts of lived experience of a particular phenomenon. In recent years there has been a growing volume of qualitative studies seeking to understand how autistic people, as well as those supporting them, experience REDs. There is value in systematically reviewing the qualitative literature in this area to draw out overall themes and use these to meaningfully inform next steps in developing more effective and neuroaffirmative ED treatments for this population. In addition, it can enable the identification of gaps in existing research. To our knowledge there are no systematic reviews of the qualitative research exploring how autistic people experience EDs.

In this review, the objective is to systematically review the qualitative research on REDs and autism in order to:

- Understand autism‐related causal and maintaining factors for REDs from the perspective of autistic people, their carers, and healthcare professionals.
- Understand barriers and facilitators for service use for this population.
- Inform future research in the development of more tailored interventions for EDs for autistic people.

## 2 Methodology

### 2.1 Protocol Registration

The protocol for this systematic review was registered in the PROSPERO database under registration number CRD42023434116.

#### 2.1.1 Search Strategy

The review methods and reporting were performed according to the Preferred Reporting Items in Systematic Review and Meta‐Analyses (PRISMA) guidelines (Page et al. 2021). The following electronic databases were searched on 11/06/23: CINAHL (Cumulative Index to Nursing and Allied Health Literature), PsycINFO, Medline, Embase, Web of Science and Global Health. Searches were run again on the 27/02/24 and no new studies were identified. The search terms and an example for one of the database searches are shown in Supporting Information S1.

#### 2.1.2 Inclusion and Exclusion Criteria

Studies were included if they met the following criteria:

- *Population:* Autistic people with lived experience of REDs, or other key stakeholders with valuable insight into experience of supporting someone with lived experience of autism and a RED. This included carers and healthcare professionals directly supporting people with this co‐occurrence. Healthcare professionals required experience of working clinically with EDs.
- *Restrictive Eating Disorder diagnosis:* REDs included AN and restrictive OSFED such as atypical AN. The study needed to report on how diagnosis was verified, such as clinical notes, self, carer, clinician reported, or validated with screening tools such as the Eating Disorder Examination Questionnaire (EDE‐Q, Fairburn, Cooper, and O’Connor 2008). In mixed ED samples, the sample needed to consist of a majority of people with REDs.
- *Autism diagnosis:* A clinical diagnosis of autism confirmed by self‐report, clinician‐report or parent‐report. Or autistic traits above cut‐off score on measures such as the Autism Quotient 10 (AQ‐10, Allison, Auyeung, and Baron‐Cohen 2012) or the Autism Diagnostic Observation Scale (ADOS, Lord et al. 2012).
- *Study type:* Findings from qualitative studies. This included qualitative components of mixed methods studies. Studies could be published or grey literature as long as they were available as completed full‐text reports, such as dissertations and preprint papers.

Commentaries, letters, conference abstracts, review articles and studies not written in English were excluded. There was no exclusion made on dates.

### 2.2 Screening and Selection of Studies

Records retrieved from the database searches were initially screened for inclusion by title and abstract. For this stage, KC screened all retrieved records and RL screened a randomly selected 10% (*n* = 123), with inter‐rater agreement of 97.6%. The full texts of identified studies were assessed for eligibility by RL and KC, with inter‐rater agreement of 92.6%. Discrepancies were discussed and resolved with TJ.

### 2.3 Quality Assessment

The Critical Assessment Skills Programme Assessment (CASP; Critical Appraisal Skills Programme 2018) for qualitative studies was applied to all identified studies. Study quality was rated as high (8–10), medium (5–7) or low (0–4). Quality assessment was conducted for all studies by KC, with 30% of studies also assessed by CGH.

Inter‐rater agreement was 86.6% and all discrepancies were discussed and resolved between KC and CGH.

### 2.4 Data Extraction and Synthesis

Descriptive data from each study were extracted including: author, year of publication and sample characteristics (number of participants, gender, ethnicity and socio‐economic status where reported).

Thematic synthesis (Thomas and Harden 2008) was used to synthesise findings from the studies. Thematic synthesis was chosen as it enables summarisation of the primary data as well as the comparison and facilitation of novel analysis and interpretation, which can lead to new insights on an issue (Sandelowski and Barroso 2007). Additionally, thematic synthesis provides a systematic and transparent approach to conducting and reporting a review of qualitative studies through its clearly defined stages. Initially the included studies were read through thoroughly, followed by extraction of all text and direct quotes relating to experiences of autism and REDs. KC and CGH then inductively coded line by line, identifying words and phrases that captured the meaning of each sentence. Themes and subthemes were initially generated independently. These were then discussed together with the wider research group to reach a final consensus.

### 2.5 Researcher Reflexivity

The research team incorporates clinical, academic and lived experience relevant to eating disorders. This review is influenced by the neuro‐affirmative stance of the team, that includes a view that autism is a form of neurodiversity, rather than a disordered developmental outcome; and that research and clinical practice in this area should be significantly influenced by engagement with the experiences of autistic people who have had restrictive eating disorders.

## 3 Results

**FIGURE 1:**
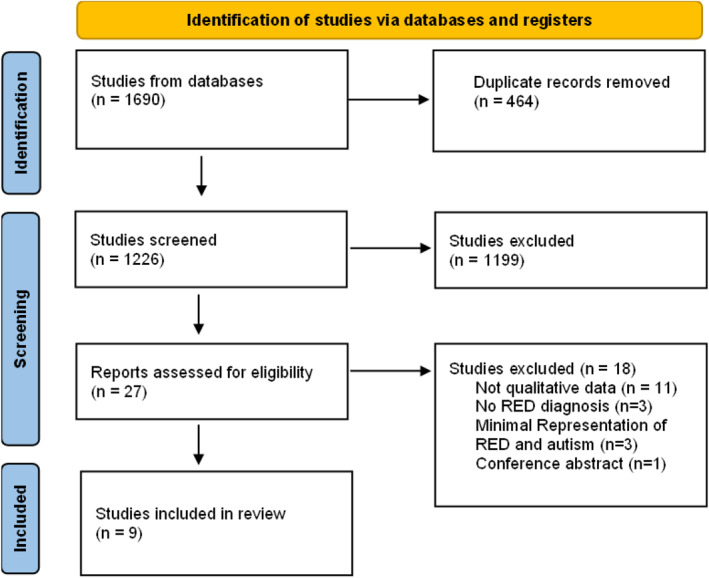
PRISMA flow diagram of the paper selection process.

The database searches returned 1690 records, of which 464 were duplicates and were removed. one thousand, two hundred twenty‐four studies were screened based on title and abstract, and 27 studies were screened based on the full text. After excluding 18 studies at full‐text screen, nine papers were included in the review (See Figure 1 for PRISMA flowchart). The study characteristics and quality scores are summarised in Table 1. A detailed summary of the critical appraisal quality scores is provided in Table 2. Eight studies were rated as high quality and one study rated as medium quality. The themes and sub‐themes are presented in Table 3.

**TABLE 1:** Study characteristics.

First author, year	Country	Aims	Sample characteristics	Autism & eating disorder characteristics	Method of qualitative data collection	Method of qualitative analysis	Quality score
Adamson et al. 2020	UK & USA	Explore the support needs of carers for loved ones with co‐occurring autism and AN and investigate carer views to see how they can be best supported.	Carers	Autism	Semi‐structured interview	Thematic analysis	High
			*n* = 10 (9 mothers and 1 father)	Clinical diagnosis of autism reported by parent			
			Gender of parents' children: Female (*n* = 10)	Eating disorder			
			Age range of parents' children (mean): 16–25 years (N/R)	Clinical diagnosis of AN reported by carer			
			Ethnicity: N/R				
			SES: N/R				
Babb et al. 2021	UK	Understand the experience of autistic women to inform service provision within eating disorder services that tailor to their needs	Autistic people:	Autism	In‐depth interviews	Thematic analysis	High
			*n* = 15	Self‐reported confirmation of a clinical diagnosis of an autism spectrum disorder.			
			Gender: Female (*n* = 15)	For autistic women taking part—score on AQ‐10 above cut off score (6 or above).			
			Age range (mean): 23–58 years (32.60)	Eating disorder			
			Ethnicity: N/R	Self‐reported confirmation of past or current experience of AN.			
			SES: N/R				
			Carers:				
			*n* = 13 (12 mothers and 1 father)				
			Gender of parents' child: Female				
			Age range of parents' child (mean): 15–31 years (24.75)				
			Ethnicity: N/R				
			SES: N/R				
			HCPs				
			*n* = 11 (6 clinical psychologists, 4 psychiatrists, 1 counselling psychologist				
Brede et al. 2020	UK	To bring together perspectives of autistic women, parents and HCPs.	Autistic people:	Autism	Semi‐structured interview	Thematic analysis	High
		To derive the first theoretical model of restrictive eating difficulties in autism	Same sample as Babb et al. 2021	Self‐reported confirmation of a clinical diagnosis of an autism spectrum disorder.			
			Carers:	For autistic women taking part—score on AQ‐10 above cut off score (6 or above).			
			Same sample as Babb et al. 2021	Eating disorder			
			HCPs	Self‐reported confirmation of past or current experience of AN			
			*n* = 16 (6 clinical psychologists, 5 psychiatrists, 1 counselling psychologist, 1 nurse, 1 speech and language therapist, 1 dietitian, 1 social worker.				
Doris et al. 2014	UK	Evaluate friendship experiences of patients with AN referred for assessment due to elevated autistic characteristics.	Patients:	Autism	Semi‐structured assessment (ADOS)	Thematic analysis	Medium
			*n* = 7	Autism traits as reported by their eating disorder HCP.			
			Gender: Female (*n* = 7)	Eating disorder			
			Age range (mean): N/R (24.8 years)	HCP confirmed current diagnosis of AN			
			Ethnicity: 7 british (6 caucasian, 1 afro‐caribbean).				
			SES: N/R				
Field et al. 2023	UK, USA & Canada	Use transparent, systematic methodology to identify research suggestions for improving eating disorder treatment for autistic women.	Experts by experience:	Autism:	Three stage delphi study. Stage one consisted of email responses to open‐ended questions.	Content analysis	High
			*n* = 20 (16 autistic people with experience of an eating disorder, 2 autistic people with no experience of an eating disorder, 2 people who have experience of an eating disorder and are not autistic).	Self‐report of lived experience			
			Gender: N/R	Eating disorder:			
			Age range (mean): N/R	Self‐report of lived experience.			
			Ethnicity: N/R				
			SES: N/R				
			HCPs				
			*n* = 19				
			Researchers				
			*n* = 7				
Kinnaird et al. 2017	UK	Explore clinicians experience of working within the autism and eating disorder comorbidity.	HCPs	n/a	Semi‐structured interview	Thematic analysis	High
			*n* = 9				
Kinnaird et al. 2019	UK	To examine the possibility of treatment adaptions for AN and autism through exploring the views and experiences of individuals with the comorbidity	Patients:	Autism:	Semi‐structured interview	Thematic analysis	High
			*n* = 13	Diagnosis of autism (*n* = 9) or had scores indicative of high autistic traits on the AQ‐10 or ADOS (*n* = 4).			
			Gender: Females (*n* = 11), non‐binary (*n* = 2)	Eating disorder:			
			Age range (mean): N/R (28.46 years)	Some of the sample came from a service where they were being treated for AN. Some of the sample self‐reported a diagnosis of AN and experience of treatment for their eating disorder.			
			Ethnicity: N/R				
			SES: N/R				
Kinnaird et al. 2021	UK	Explore the support needs of carers for loved ones with co‐occurring autism and AN and investigate carer views to see how they can be best supported.	Carers:	Carers self‐reported current caring responsibilities for someone with diagnoses of both an eating disorder and autism.	Semi‐structured interview	Thematic analysis	High
			*n* = 11 (three fathers and eight mothers)	One carer's child did not yet have an autism diagnosis but had scored positively on the ADOS.			
			Gender of parents' child: Female (*n* = 8), males (*n =* 3)	Carers reported having children with a diagnosis of AN restrictive subtype (*n* = 9), AN binge‐purge subtype (*n* = 1) and BED (*n* = 1).			
			Age range of parents' child (mean): 12–36 years (23.00)				
			Ethnicity: N/R				
			SES: N/R				
Nimbley et al. 2023	UK	Explore social and sensory differences across autistic and non‐autistic adults with current lifetime history of AN and their parent/carer.	Autistic people:	Autism:	Dyadic interview	Interpretive phenomenological analysis	High
			*n* = 7	Self‐reported			
			Gender: Females (*n* = 6), males (*n* = 1)	Eating disorder:			
			Age range (mean): N/R (24.14 years)	Have received a clinical diagnosis of either AN (including atypical AN).			
			Ethnicity: White (*n* = 7)				
			SES: Student (*n* = 2), unemployed (*n* = 2), part‐time employment (*n* = 1), full‐time employment (*n* = 1)				
			Carers:				
			*n* = 7 (seven mothers)				
			Gender of parents' child: Females (*n* = 6), males (*n* = 1)				
			Age range of parents' child (mean): N/R (24.14 years)				
			Ethnicity: White (*n* = 7)				
			SES: Full‐time employment (*n* = 3), self‐employed (*n* = 1), full‐time carer (*n* = 1), part‐time employment (*n* = 1), retired (*n* = 1)				

Abbreviations: ADOS = Autism Diagnostic Observation Schedule; AN = Anorexia Nervosa; AQ‐10 = Autism Spectrum Quotient (self‐report measure); ARFID = Avoidant Restrictive Food Intake Disorder; BED = Binge Eating Disorder; BN = Bulimia Nervosa; HCP = Health Care Professionals; N/R = not reported; SES = socio‐economic status; UK = United Kingdom; USA = United States of America.

**TABLE 2:** Critical appraisal skills program (CASP) summary.

CASP	Adamson et al. (2020)	Babb et al. (2021)	Brede et al. (2020)	Doris et al. (2014)	Field et al. (2023)	Kinnaird et al. (2017)	Kinnaird et al. (2019)	Kinnaird et al. (2021)	Nimbley et al. (2023)
**Validity**									
Aims	Yes	Yes	Yes	Yes	Yes	Yes	Yes	Yes	Yes
Method	Yes	Yes	Yes	Yes	Yes	Yes	Yes	Yes	Yes
Research design	Partial	Yes	Yes	Partial	Yes	Partial	Yes	Yes	Yes
Recruitment	Yes	Yes	Yes	Yes	Yes	Yes	Yes	Yes	Yes
Data collection	Yes	Yes	Yes	Partial	Yes	Yes	Yes	Yes	Yes
Reflexivity	Partial	Yes	Yes	No	No	No	No	No	Yes
**Results**									
Ethics	Yes	Yes	Yes	Yes	Yes	Yes	Yes	Yes	Yes
Analysis	Yes	Yes	Yes	Yes	Yes	Yes	Partial	Yes	Yes
Findings	Yes	Yes	Yes	Yes	Yes	Yes	Yes	Yes	Yes
**Value**									
Value of research	Yes	Yes	Yes	Yes	Yes	Yes	Yes	Yes	Yes
**Total**	8	10	9	7	9	8	8	9	10
**Rating**	High	High	High	Medium	High	High	High	High	High

NoteLow = 0–4, Medium = 5–7, High = 8–10

**TABLE 3:** Themes and sub‐themes from meta‐synthesis.

Theme	Sub‐theme	Studies
Diverse mechanisms linking autism and restrictive eating	Sensory needs	Adamson et al. (2020), Babb et al. (2021)/Brede et al. (2020), Field et al. (2023), Kinnaird et al. (2019, 2017), Kinnaird et al. (2021), Nimbley et al. (2023),
	Emotions	Babb et al. (2021)/Brede et al. (2020), Kinnaird et al. (2019, 2017), Nimbley et al. (2023),
	Thinking styles	Adamson et al. (2020), Babb et al. (2021)/Brede et al. (2020), Field et al. (2023), Kinnaird et al. (2019, 2017), Kinnaird et al. (2021),
	Social and communication difficulties	Adamson et al. (2020), Babb et al. (2021)/Brede et al. (2020), doris et al. (2014), Field et al. (2023), Kinnaird et al. (2019), Nimbley et al. (2023)
	The role of weight and shape	Babb et al. (2021)/Brede et al. (2020), Kinnaird et al. (2019), Nimbley et al. (2023)
The journey to self‐understanding	Lacking a sense of identity	Brede et al. (2020), Field et al. (2023), Kinnaird et al. (2019), Nimbley et al. (2023)
	Value of the autism diagnosis	Brede et al. (2020), Kinnaird et al. (2017) Kinnaird et al. (2019, 2021), Nimbley et al. (2023),
	Delays to the autism diagnosis	Adamson et al. (2020), Babb et al. (2021)/Brede et al. (2020), Kinnaird et al. (2019, 2017), Kinnaird et al. (2021)
Experience of eating disorder services	Feeling misunderstood by ED professionals	Adamson et al. (2020), Babb et al. (2021), Field et al. (2023), Kinnaird et al. (2017), Kinnaird et al. (2019, 2021), Nimbley et al. (2023)
	Clinicians' uncertainty	Babb et al. (2021), Field et al. (2023), Kinnaird et al. (2017)
	Feeling let down by current psychological treatment options	Adamson et al. (2020), Babb et al. (2021), Field et al. (2023), Kinnaird et al. (2019, 2021).
	Positive experiences once autism was recognised	Adamson et al. (2020), Babb et al. (2021)
	Increased carer burden	Adamson et al. (2020), Kinnaird et al. (2021)
Treatment adaptations	Sensory adaptations	Adamson et al. (2020), Babb et al. (2021), Field et al. (2023), Kinnaird et al. (2019, 2017), Kinnaird et al. (2021)
	Broader communication	Adamson et al. 2020), Babb et al. (2021), Field et al. (2023), Kinnaird et al. (2019, 2017) Nimbley et al. (2023)
	Being mindful of different thinking styles	Babb et al. (2021), Field et al. (2023), Kinnaird et al. (2019, 2017),
	Having the time to build trust	Adamson et al. (2020), Field et al. (2023), Kinnaird et al. (2019, 2021)
	Approach change in a neuroaffirmative way	Field et al. (2023), Kinnaird et al. (2019, 2017)
	One size does not fit all	Adamson et al. (2020), Babb et al. (2021), Field et al. (2023), Kinnaird et al. (2019, 2017), Kinnaird et al. (2021)
	Building adaptive coping skills	Babb et al. (2021)/Brede et al. (2020), doris et al. (2014), Field et al. (2023), Kinnaird et al. (2017), Kinnaird et al. (2019)
	Supporting loved ones	Adamson et al. (2020), Field et al. (2023), Kinnaird et al. (2017, 2021)
	Increased knowledge and skills in autism through training	Adamson et al. (2020), Field et al. (2023), Kinnaird et al. (2019, 2017), Kinnaird et al. (2021).

## 4 Study Characteristics

All studies were based in the United Kingdom (UK), with two studies including participants from the United States of America (USA) and Canada. The only RED that was examined in studies was AN. Two of the studies (Babb et al. 2021; Brede et al. 2020) had significant overlap in a shared sample, with all autistic participants and carers being the same across the two studies, and overlaps between the two for the healthcare professionals sample (confirmed via personal communication with authors). The interviews were separately analysed for each study with a different focus in their analysis. To account for this when coding, if a theme arose in both studies they were counted as one combined study, to avoid giving double weighting to the same themes arising in both. This is reflected in Table 3, where themes that arose in both studies are referenced as ‘Babb et al. 2021/Brede et al. 2020’, rather than separately.

Six studies included the experiences of autistic people (or those with a high number of autistic traits) with AN, representing the views of 58 autistic adults with a RED (39 females, two non‐binary people, one male, and then 16 people from one study where gender was not reported). Five studies included the experiences of parents/carers (*n* = 40), with these being parents of both adolescents and adults. Four studies included the views of ED healthcare professionals (*n* = 32). No study included the direct experience of adolescents themselves, although some of the parents were caring for adolescents. Two studies reported ethnicity of their sample (Doris et al. 2014; Nimbley et al. 2023). Nimbley et al. 2023 also reported on socioeconomic status of participants. There was variation in how autism or eating disorder status was confirmed. Most studies took the approach of participants self‐confirming that they had received an autism diagnosis and ED (or cared for someone with an autism diagnosis and ED). In addition to this, some studies also asked autistic participants to complete a self‐report screening measure, such as the AQ‐10.

### 4.1 Theme 1: Diverse Mechanisms Linking Autism and Restrictive Eating

A wide range of autism‐related difficulties were highlighted as making significant contributions to the development and perpetuation of autistic people's ED. Restrictive eating was often framed as a way of coping directly with autism‐related difficulties, and autism was commonly reported to make recovery from the ED more challenging in other ways. Sub‐themes are described below, with sub‐theme names appearing in bold text.

**Sensory needs**, particularly hyper‐sensitivity, were noted as being a key factor in the aetiology of the ED. This came up in the context of both the overwhelming sensory properties of food such as taste, texture and smell, as well as feeling overstimulated by certain environments, making it harder to eat. One autistic person described sensory sensitivity as ‘a full body response to it as opposed to oh I like that smell, I don't like that smell’ (Nimbley et al. 2023). Parents also reflected on the impact of sensory sensitivities on their child's ability to cope by noting how sensory overload would often trigger a meltdown. People spoke about escaping this sense of overwhelm through restrictive eating or excessive exercise. Both of these acted as a way to reduce these aversive experiences or to feel in control of something when feeling overwhelmed. Having a set and selective way of eating at these times was helpful, being described as ‘less mental noise’ (Nimbley et al. 2023).

Difficulties with interoceptive awareness were also raised, leading to challenges with recognising internal sensations such as emotions, hunger, thirst and digestion. This left people vulnerable to not eating enough or avoiding eating to avoid these internal experiences that felt confusing or aversive (Brede et al. 2020; Nimbley et al. 2023). Linked to interoception, many highlighted the longstanding difficulties identifying, regulating and communicating their **emotions**, and feeling overwhelmed as a result of this (Brede et al. 2020; Kinnaird et al. 2017; Nimbley et al. 2023). Autistic people in Nimbley et al. (2023) described a heightened sensitivity to emotions, possibly linked to interoceptive sensitivities, but not being able to name or regulate these feelings. Restrictive eating again was described as a way to numb and distract from the overwhelm. For example, avoidance of ‘embarrassing’ meltdowns or channelling their anxiety by focussing worry on food and nothing else (Brede et al. 2020).

Different **thinking styles** were described across the studies, with an increased tendency for autistic people to be literal, rigid thinkers with a more detail‐oriented and narrow focus of attention. This included a tendency to have intense and very focused interests that could tip into becoming more obsessive in nature. This different way of thinking and focused attention was thought to leave people more vulnerable to developing rules around eating and food and then difficulty in shifting their focus away from these rules once established (Brede et al. 2020; Kinnaird et al. 2017). For example, people talked about taking public health advice around diet and exercise in a very literal, ‘all or nothing’ way that then might escalate into AN. Similarly, a strong preference for routine and sameness was often applied to eating: ‘they might have the same food everyday or have three different foods that they eat and they can't bear to try anything else, they just can't bear it and it's quite hard to shift’ (Kinnaird et al. 2017). It was also referenced as a barrier to engagement with professionals who might misinterpret literalness as rudeness (Nimbley et al. 2023).

**Social and communication difficulties**, particularly with peers, were named as being a causal factor for developing AN, with these being lifelong difficulties rather than a consequence of the AN. Carers spoke about friendships becoming much more difficult for their daughters to navigate as they moved out of childhood, where friendships were organised more by parents, and into adolescence where their child had to navigate initiating and maintaining friendships more independently (Adamson et al. 2020). Autistic women spoke about restrictive eating as a way to cope, distract and numb themselves when experiencing loneliness, bullying and finding friendships hard. The AN was described as something else to focus on, as well as a way to fit in (Brede et al. 2020). People described feeling different and therefore ‘defective’, especially in relation to their peers, leading to feelings of social isolation and alienation. This feeling of not being worthy could translate into feeling like they did not deserve to eat (Nimbley et al. 2023). Autistic people and parents reflected that at a time when the autism was unrecognised and the autistic person felt different from their peers, some focused on **weight and shape concerns**. This was sometimes described as an attempt to try and feel accepted by others, because societal messages around them emphasised the value of being thin. They then turned their focus to weight loss to try and resolve feeling socially isolated and to fit in. For others they were looking to make sense of why they felt different to others and concluded that it must relate to their weight or shape. People across studies also noted that weight and shape concerns were not always driving the restrictive eating, instead being more to do with difficulties captured by the other ‘diverse mechanisms linking autism and restrictive eating’ sub‐themes.

### 4.2 Theme 2: Journey to Self‐Understanding

Many autistic individuals described **lacking a sense of identity**, feeling different and not fitting in as central to the development of their ED, and linking these experiences to unrecognised autism. Many of the participants and children of carers were diagnosed with the ED before they were diagnosed with autism. In Babb et al. (2021) the average age of autism diagnosis was 12 years higher than the average age of AN diagnosis. AN was described as providing a sense of identity, allowing them to immerse themselves in an ED, similar to an autistic special interest or passion (Brede et al. 2020). AN also became a way to manage feeling different in the context of not knowing they were autistic (Kinnaird et al. 2019; Nimbley et al. 2023). This connected to the **value of the autism diagnosis** in enabling movement toward more self‐understanding. This included being able to make connections between how unrecognised autism may be contributing to their ED. The diagnosis then gave them and their loved ones more confidence to advocate for their own needs, sometimes leading to more appropriate support (Kinnaird et al. 2019). Many described significant **delays to the autism diagnosis** due to multiple barriers to accessing autism assessment, with some then accessing this privately due to a long waiting list. One carer commented on their daughter not being considered for assessment because ‘for years they have been diagnosing girls on the boys criteria which is hopeless’ (Adamson et al. 2020). Clinicians provided some insight into some of the reasons delays might occur. They referenced there often being no clear or common pathways for referrals for autism assessments in ED services (Kinnaird et al. 2017), adding to the uncertainty of how to proceed when working with someone who was autistic or possibly unrecognised autistic. Some questioned the timing of doing an assessment and whether diagnosis would be helpful (Kinnaird et al. 2017). Others spoke about delaying assessment until the patient was weight restored due to the possibility of starvation mimicking some autistic features and therefore reducing the validity of assessment (Kinnaird et al. 2017).

### 4.3 Theme 3: Experiences of Eating Disorder Services

Many participants across the studies reflected on **feeling misunderstood by ED professionals**. This often arose in the context of a perceived insufficient awareness of autistic mechanisms for restrictive eating and how to adapt appropriately for these in treatment. Autistic traits were often interpreted under traditional approaches focussing on weight and shape driven behaviours. Autism related difficulties could be misinterpreted as AN behaviour and labelled as ‘naughty’ or ‘uncooperative’, for example fidgeting/stimming or socio‐communication differences being misinterpreted as rudeness (Babb et al. 2021; Kinnaird et al. 2019; Nimbley et al. 2023). This left autistic individuals feeling misunderstood. For autistic people, this lack of awareness and, at times, dismissal of their autism further contributed to sensory overwhelm, exacerbating the ED (Field et al. 2023; Kinnaird et al. 2019; Babb et al. 2021; Brede et al. 2020). In some cases, this would lead to disengagement from treatment and individuals reported being labelled as ‘too complex’ by healthcare professionals (Field et al. 2023; Babb et al. 2021; Nimbley et al. 2023). This was also reflected by **clinicians' uncertainty** describing the difficulties of disentangling AN and autism (Babb et al. 2021; Kinnaird et al. 2017), particularly in terms of set thinking patterns and routines impacting ED behaviour. In Kinnaird et al. (2017), 60% of clinicians reported a lack of confidence or experience in treating autistic patients, highlighting the inconsistency of training and awareness of the relationship between autism and EDs. Clinicians described noticing the difference in autistic communication style and noting its impact on building a therapeutic relationship, but not being sure what to do about it. ‘It's really hard to get communication going’ (Kinnaird et al. 2017). Autistic people and their carers reported **feeling let down by current psychological treatment options** for the ED (Adamson et al. 2020; Kinnaird et al. 2019). This included evidence‐based therapies for adolescent AN such as Cognitive Behavioural Therapy for Eating Disorders (CBT‐ED) and Eating Disorder focused Family Therapy (FT‐ED). CBT‐ED was criticised by some autistic people and clinicians as holding certain assumptions about baseline skills, for example the ability to easily generalise skills from one situation to another or the ability to identify thoughts and emotions with relative ease (Babb et al. 2021; Kinnaird et al. 2017). Carers in Kinnaird et al. (2021) described feeling like FT‐ED was not appropriate for autistic people, specifying difficulties with capacity for abstract thought and demand avoidance. Across both therapies, participants commented on typical time frames as insufficient, needing the flexibility for longer treatment.

People also shared **positive experiences once autism was recognised** (Babb et al. 2021; Adamson et al. 2020). This was not everyone's experience, with others reporting finding it harder to access ED support once the autism diagnosis was made or experiencing refusal for ED input because of being autistic. The positive experiences depended on the service and allocated therapist having knowledge of autism and its potential influence on eating. In Adamson et al. (2020) carers reflected that the clinicians who took the time to build a relationship with their child made a positive impact in the treatment. Structured groups such as Dialectical Behavioural Therapy had more positive feedback (Babb et al. 2021) with its focus on emotion regulation. Occupational Therapy was also noted for its helpfulness due to its more practical and goal‐focused orientation.

Carers spoke of a lack of specific support for autism comorbidity and having to set up their own support groups and networks online to manage **increased carer burden:** ‘None of the (eating disorder) workshops we attended addressed those extra needs’ (Adamson et al. 2020); “(The community treatment team) kept talking about ‘riding the wave of anorexia’ but it wasn't a wave, it was a full‐on tsunami” (Kinnaird et al. 2021). They described having to take an active role in advocating for their child to receive appropriate care, compensating for a lack of appropriate support in services. This in turn led to an increase in caring responsibilities, with consequences such as taking early retirement, stopping work, as well as increased financial burden through seeking private treatment (Kinnaird et al. 2021).

### 4.4 Theme 4: Treatment Adaptations

Based on many of the challenges covered by earlier themes, participants from all studies reported on what treatment adaptations would improve the experience of getting support for a RED in the context of also being autistic. **Sensory adaptations** were highlighted as crucial. This included environmental adaptations, for example reducing noise in services by having soft close doors (Adamson et al. 2020; Babb et al. 2021, Field et al. 2023), as well as adapting meals plans to account for sensory differences (Adamson et al. 2020, Field et al. 2023; Kinnaird et al. 2021). Consideration of **broader communication options** was also named in different ways. This included use of written summaries (Babb et al. 2021; Field et al. 2023), focused questions such as ‘what did you do today’ rather than ‘how are you?’ (Babb et al. 2021) and creating communication passports, which are brief individual documents designed to provide healthcare professionals with important information about the person when supporting or treating them. Helping people to feel more comfortable in expressing themselves by reducing over‐stimulation was also named.

**Being mindful of different thinking styles,** such as literal thinking, all or nothing thinking, and a strong preference for detail and predictability were also mentioned as potential adaptations. This could also include supporting someone's preference for sameness by maintaining a routine with appointment time and location (Kinnaird et al. 2017). Where helpful, this may mean supporting someone to become more flexible where cognitive rigidity is a barrier to their goals (Kinnaird et al. 2019). Participants also shared the importance of **having the time to build trust** before expecting someone to do any challenging psychological work. In one study carers spoke about how clinicians who focused on this at the start made a significant positive impact in how their child's treatment progressed (Adamson et al. 2020). Participants highlighted the importance of recognising a need for longer treatment duration, in order to establish rapport and to have space to work through difficulties with change and cognitive flexibility (Adamson et al. 2020; Kinnaird et al. 2019, 2021).

Whilst change was reported as important, participants underlined needing to **approach change in a neuro‐affirmative way** by not setting targets which aim to change core parts of the individual. Instead, people initially wanted support to understand their experiences in relation to autism and the ED (Field et al. 2023). Participants in Field et al. (2023) also highlighted the need to know more about how autistic people define recovery from an ED. Linked to this, there was also the importance and protective effect of meeting like‐minded people to facilitate feeling accepted, with one person reflecting that this did not happen until she met others who were ‘wired the same way’ on an inpatient ward. Others were explicit that **‘one size does not fit all’** when it comes to what works in treatment (Field et al. 2023) and that it is important to not just focus on food, weight and body image (Babb et al. 2021).

Support in **building adaptive coping skills** for autism‐related difficulties was proposed as a process toward removing the need to turn to restrictive eating and excessive exercise to self‐regulate (Field et al. 2023). Possible coping skills highlighted included the development of emotional literacy and regulation (Kinnaird et al. 2017, 2019), or others might benefit most from friendship focused interventions (Doris et al. 2014). Considering the wider system, there was also the need for **supporting loved ones** such as carers, parents and wider family. Suggestions included peer support groups and support from others with lived experience of caring for someone with both AN and autism (Kinnaird et al. 2021). Participants wanted to have more psychoeducation around co‐occurring autism and EDs, as well as more communication from clinicians and to be more involved with their treatment.

Finally, in order to deliver these adaptations consistently, participants across all three groups highlighted the importance of staff having **increased knowledge and skills in autism through training**. It was noted that this should include specialist training about how autism‐related differences can make eating difficult (Kinnaird et al. 2021). People also highlighted the need to include lived experience in the development and delivery of training for staff (Field et al. 2023; Kinnaird et al. 2021).

## 5 Discussion

Our review highlights that across the studies, autistic people, parents and healthcare professionals consistently report that autism plays a significant role in the development and maintenance of AN. They also note that these autism‐related difficulties are not reliably integrated into the formulation and treatment of the ED. A wide range of possible areas for treatment adaptation and improvement were identified.

When considering the variety of reasons why an autistic person might be vulnerable to developing an ED, many raised how overwhelming life can be as an autistic person living in a world primarily designed for non‐autistic people. This is perhaps even more the case for the people in these studies, almost all of whom were women, as most of them did not know they were autistic when they developed AN. Research highlights gender differences in how autism can be expressed, often referred to as the female autism phenotype (Gould and Ashton‐Smith 2011; Hull, Petrides, and Mandy 2020). This has led to women being more at risk of being undiagnosed, thought to be partly due to a male bias in current screening and diagnostic tools (Dworzynski et al. 2012; Loomes, Hull, and Mandy 2017). Our findings echo this, with some participants' autism reportedly being missed due to professionals not being aware of how autism can present differently in females.

In our review, people named how their autism, often unrecognised, left them with a feeling of not fitting in and that they experienced many invalidating life experiences in their childhood and adolescence. A common thread across the studies described people turning to restrictive eating and AN as a way to cope with these experiences. For some this was a way to create routine and predictability in an overwhelming and unpredictable world. For others it was a way to reduce sensory overwhelm by avoiding certain foods and environments. Several participants described weight loss as a way of trying to fit in with neurotypical peers and societal messages that thinness should be valued. The transition from being younger and having social interactions scaffolded by parents, to becoming older and the adolescent being expected to take more responsibility for initiating and maintaining friendships was highlighted as a vulnerable time for the ED to develop. This coincides with the onset of puberty, which is a significant change, physically, socially and emotionally, and was also noted as a moderating factor for eating difficulties (Brede et al. 2020). Our findings support previous research arguing for the need to improve timely access to assessment and post‐diagnostic support (Bargiela, Steward, and Mandy 2016), the latter also being raised by some as non‐existent or very minimal.

## 6 Strengths and Limitations

In terms of strengths, this is the first thematic synthesis of the autistic experience of restrictive EDs. We adhered to PRISMA guidelines and involved several reviewers across the search strategy formulation, study selection, quality assessment and thematic synthesis. A limitation of the review is that participant samples were predominantly adult women with experience of AN, and the studies that did report on ethnicity reported the sample being predominantly white. Thus, our review highlights gaps in our knowledge in terms of the experiences of children and adolescents, men and non‐binary people, and people from minoritised ethnicities. A further limitation is that the qualitative data synthesised came from several data collections sources, including interviews, surveys and assessments. This means that there were differences in the richness of the data, with interview studies providing the greatest depth and nuance.

A possible limitation of our review criteria is that we included studies where participants self‐confirmed having a clinical diagnosis of both autism and an eating disorder. This means that the diagnostic status of the research participants with lived experience of autism and eating disorders is less certain than would have been the case had researchers only included participants where they could show evidence of clear proof of clinical diagnosis. Therefore, we have made an assumption by accepting self‐confirmed clinical diagnosis that participants have received an assessment and diagnosis via a clinical service. At the same time there is an increasing move toward including those who self‐identify as autistic, with some research beginning to include self‐diagnosed autistic people in research. This is partly to be more inclusive and recognise the significant barriers to referrals for autism diagnostic assessments (Overton et al. 2023). This mirrors the finding from our systematic review, with people describing significant delays in receiving their autism diagnosis.

### 6.1 Implications for Future Research and Clinical Practice

Adaptations described by clinicians in the Kinnaird et al. (2017) paper reflected many of the adaptation suggestions that autistic participants and their parents/carers said they wanted more of (e.g. sensory adaptations, thoughtfulness around autistic thinking styles, supporting with emotional literacy). This indicates that these adaptations are taking place in some ED services and not in others. Future research could develop and evaluate the effectiveness of training packages for clinicians, focussing on autism, and ensuring these are co‐produced with people with lived experience of autism and EDs. Furthermore, there is value in formally testing the treatment adaptations that have been suggested across the qualitative studies, in order to evaluate whether or not they lead to improved treatment outcomes.

The absence of qualitative studies with adolescents demonstrates a need to systematically gather the views of how autistic children and adolescents experience the development of restrictive EDs and current treatments. This is particularly important in terms of their experience of treatment, with treatment guidelines for adolescents and adults being different (NICE 2017). The primary evidence‐based treatment for AN in children is FT‐ED. In one of the qualitative studies, parents referenced their child receiving family therapy and not finding this helpful (Kinnaird et al. 2021). Further qualitative research is needed to understand experiences of FT‐ED in autistic young people and their families, whilst quantitative research is needed to investigate treatment outcomes for this group, which may be poorer (Nielsen et al. 2015) and therefore require adaptation.

## 7 Conclusion

This systematic review synthesised current qualitative literature on the autistic experience of REDs. We identified key considerations for conceptualising how autistic people experience AN, with autism‐related differences being reported as playing a significant role in the development and maintenance of AN. As maintaining factors are often a target in treatment, our findings also point to the need for the development and evaluation of novel or adapted interventions for autistic individuals experiencing an eating disorder. The synthesis also highlighted gaps in research, with a need for qualitative research with more diverse samples of people with co‐occurring autism and REDs. This includes autistic people who are under 18, as well as more gender diversity, as the current studies predominantly captured the adult female experience. Our findings also underline the importance of consistently establishing and delivering training on autism to ED clinicians in order to provide reliably high‐quality care to this population.

## Ethics Statement

The authors have nothing to report.

## Consent

The authors have nothing to report.

## Conflicts of Interest

The authors declare no conflicts of interest.

## Permission to Reproduce Material From Other Sources

Not Applicable.

## Patient Consent Statement

Not applicable.

## Supporting information

Supporting Information S1

## Data Availability

Data sharing is not applicable to this article as no new data were created or analysed in this study.
