# Supplementary material for: Understanding the Autistic Experience of Restrictive Eating Disorders—A Systematic Review and Qualitative‐Synthesis
Source: Eur Eat Disord Rev. 2025 Mar 5;33(4):800–14. doi: 10.1002/erv.3181 (PMC12171672; doi:10.1002/erv.3181)
Supplement: Supplementary file 1 — Supporting Information S1 [file ERV-33-800-s001.docx]

**Supplemental 1: Search strategy, search terms and example of database search**

**Search Strategy:**

| Search strategy | Concept | Facet analysis | Example search terms |
| --- | --- | --- | --- |
| Population | Autism | Autism spectrum disorder  ASD  ASC | Autis*.tw. OR  ASD.tw. OR  ASC.tw. OR  Subject headings: autism/ |
| Exposure | Eating disorder | Eating disorder  Restrictive eating disorder  Anorexia nervosa  ARFID  Avoidant restrictive food intake disorder  Other specified feeding or eating disorders  OSFED | ‘eating disorder’ OR  ‘restrictive eating OR disorder’ OR  Anorexi* OR  ‘Avoidant restrictive food intake disorder’ OR  “other specified feeding or eating disorder” OR  OSFED OR  Subject headings:  Eating Disorder/ OR  Anorexia nervosa/ OR |
| Outcome | Experience | Experience  Outcome  Recovery | Experience* OR  Outcome* OR  Recover* OR  Subject headings:  Experience/ OR  Outcome/ OR  Mental health recovery/ |

**Search Terms:**

The search terms ‘autism’ (‘autis*.tw’ OR ‘ASD.tw’ OR ‘ASC.tw’) AND ‘restrictive eating disorders’ (‘eating disorder’ OR ‘restrictive eating OR disorder’ OR Anorexi* OR ‘Avoidant restrictive food intake disorder’ OR “other specified feeding or eating disorder” OR OSFED) AND ‘experience’ (‘experience*’ OR ‘outcome*’ OR ‘recover*’). Limits ‘English language’ were applied.

**Search strategy example:**

Database: APA PsycInfo <1806 to June Week 1 2023>
Search Strategy:
--------------------------------------------------------------------------------
1     exp Autism Spectrum Disorders/ (54502)
2     autis*.tw. (65634)
3     ASD.tw. (25303)
4     ASC.tw. (1047)
5     1 or 2 or 3 or 4 (68990)
6     exp Eating Disorders/ (35308)
7     "eating disorder*.".tw. (30071)
8     "restrictive eating disorder*.".tw. (110)
9     exp Anorexia Nervosa/ (12451)
10     anorexi*.tw. (18970)
11     "avoidant restrictive food intake disorder*".tw. (341)
12     ARFID.tw. (251)
13     "other specified feeding or eating disorder".tw. (64)
14     OSFED.tw. (76)
15     6 or 7 or 8 or 9 or 10 or 11 or 12 or 13 or 14 (45982)
16     outcome*.tw. (489440)
17     experience*.tw. (762863)
18     recover*.tw. (97054)
19     exp "Recovery (Disorders)"/ (14923)
20     exp "Treatment Process and Outcome Measures"/ (941)
21     16 or 17 or 18 or 19 or 20 (1227569)
22     5 and 15 and 21 (274)
23     limit 22 to (human and english language) (244)
